# Tug-of-Peace: Visual Rivalry and Atypical Visual Motion Processing in MECP2 Duplication Syndrome of Autism

**DOI:** 10.1523/ENEURO.0102-23.2023

**Published:** 2024-01-16

**Authors:** Daria Bogatova, Stelios M. Smirnakis, Ganna Palagina

**Affiliations:** 1 Department of Neurology, Brigham and Women’s Hospital, Boston, MA 02115; 2 Department of Biology, Boston University, Boston, MA 02115; 3 Harvard Medical School, Boston, MA 02115; 4 Jamaica Plain Veterans Affairs Hospital, Boston, MA 02130

**Keywords:** autism, bistable perception, MECP2 duplication, visual motion perception, visual rivalry

## Abstract

Extracting common patterns of neural circuit computations in the autism spectrum and confirming them as a cause of specific core traits of autism is the first step toward identifying cell-level and circuit-level targets for effective clinical intervention. Studies in humans with autism have identified functional links and common anatomic substrates between core restricted behavioral repertoire, cognitive rigidity, and overstability of visual percepts during visual rivalry. To study these processes with single-cell precision and comprehensive neuronal population coverage, we developed the visual bistable perception paradigm for mice based on ambiguous moving plaid patterns consisting of two transparent gratings drifting at an angle of 120°. This results in spontaneous reversals of the perception between local component motion (plaid perceived as two separate moving grating components) and integrated global pattern motion (plaid perceived as a fused moving texture). This robust paradigm does not depend on the explicit report of the mouse, since the direction of the optokinetic nystagmus (OKN) is used to infer the dominant percept. Using this paradigm, we found that the rate of perceptual reversals between global and local motion interpretations is reduced in the methyl-CpG-binding protein 2 duplication syndrome (MECP2-ds) mouse model of autism. Moreover, the stability of local motion percepts is greatly increased in MECP2-ds mice at the expense of global motion percepts. Thus, our model reproduces a subclass of the core features in human autism (reduced rate of visual rivalry and atypical perception of visual motion). This further offers a well-controlled approach for dissecting neuronal circuits underlying these core features.

## Significance Statement

Autism is a disorder of distributed computations, spanning low-level sensation and high-level sensorimotor integration, decision-making and social cognition. A distributed computation involving both low-level sensory and high-level executive processes, visual rivalry represents a potential candidate approach for the study of autism. We developed and applied the monocular rivalry paradigm based on competition between local and global visual motion in the mouse model of monogenic autism - MECP2 duplication syndrome. MECP2 duplication mice show slowed visual rivalry and favor local over global motion interpretation of the stimulus. This recapitulates the phenotype of human idiopathic autism and offers a way to dissect the circuit of altered visual motion processing and visual rivalry in autism using mouse models of autism.

## Introduction

Autism is a group of neurodevelopmental disorders traditionally conceptualized as impairments of high-level cognitive functions leading to deficient social communication and repetitive restricted behavioral repertoire. These high-level features are accompanied by a distinct perceptual style focusing on the fine details of the environment rather than globally integrated scenes (C.E. Robertson and Baron-Cohen, 2017; Van der Hallen et al., 2019). Even before social deficits become evident, over 90% of individuals with autism experience altered sensation and atypical sensory perception that affect every sensory modality (Simmons et al., 2009; Grzadzinski et al., 2013; A.E. Robertson and Simmons, 2015; C.E. Robertson and Baron-Cohen, 2017; Van der Hallen et al., 2019). These traits are diagnostic feature of autism and a part of restricted repetitive behaviors (American Psychiatric Association, 2013). Importantly, the expression of core traits is remarkably diverse across individuals with autism, affecting all aspects of interaction with physical and social environments (Shafritz et al., 2008; Baron-Cohen et al., 2009; Lawson et al., 2015; C.E. Robertson and Baron-Cohen, 2017; Van der Hallen et al., 2019; Bolton et al., 2020; Uddin, 2021). Thus, it is crucial to develop and use paradigms that can assess the mechanisms of and interactions between low-level (sensation, stereotypies) and high-level (social cognition, cognitive rigidity) traits of autism.

In this work, we apply a bistable visual perception paradigm to study the mouse model of methyl-CpG-binding protein 2 duplication syndrome (MECP2-ds; Collins et al., 2004; Ramocki et al., 2010), a syndromic autism spectrum disorder (ASD) caused by genomic duplication of MECP2 (Ramocki et al., 2010) that exhibits 100% penetrance in males. In humans, MECP2 duplication syndrome displays all core features of idiopathic autism (Peters et al., 2013; Ta et al., 2022). Analogously, MECP2-ds mice display repetitive stereotyped behaviors, altered vocalizations, increased anxiety, motor savant phenotype and over-reliable visual responses (Collins et al., 2004; Samaco et al., 2012; Jiang et al., 2013; Sztainberg et al., 2015; Ash et al., 2017, 2021a, b, 2022; Zhang et al., 2017; Zhou et al., 2019).

Bistable visual perception paradigms are a natural choice for studying autistic brains. First, the dynamics of visual rivalry are altered in idiopathic human autism, with subjects showing a decreased rate of perceptual reversals (C.E. Robertson et al., 2013; Spiegel et al., 2019). Second, visual rivalry is a distributed computation involving low-level sensory cortical areas as well as high-level association areas, such as the secondary motor cortex and prefrontal cortex (Leopold and Logothetis, 1996, 1999; Kleinschmidt et al., 1998; Lumer et al., 1998; Lumer and Rees, 1999; Knapen et al., 2011). Thus, its dynamics are based on stimulus representation subnetworks in the early visual cortex as well as visuomotor areas and high-level cognition nonsensory subnetworks of higher-order cortical areas. As a result, it is a suitable candidate method to evaluate both low-level and high-level sensory processing dysfunction, and its interaction with social cognition and decision-making. Indeed, in human autism slower rate of bistable alternations was shown to share an anatomic substrate with general cognitive rigidity, and binocular rivalry phenotype predicts the severity of social phenotype and the diagnosis of ASD (Spiegel et al., 2019; T. Watanabe et al., 2019).

Importantly, it was suggested that the dynamics of the visual rivalry are dependent on brain-wide excitatory-inhibitory balance, a process that is also proposed to be altered in autism (Zhao et al., 2021). Finally, our visual rivalry paradigm utilizes a bistable moving plaid, in which the subject’s perception switches between the local motion-based, “transparent” interpretation of the stimulus versus the global motion-based, “coherent” interpretation. Thus, our paradigm also offers the additional advantage of exploring another core trait of autistic brains: atypical processing of visual motion and detail-oriented sensory processing style (C.E. Robertson and Baron-Cohen, 2017; Van der Hallen et al., 2019).

## Materials and Methods

### Animals

All experiments and animal procedures were performed in accordance with guidelines of the National Institutes of Health for the care and use of laboratory animals and were approved by the Brigham and Women’s Hospital Institution Animal Care and Use Committee (IACUC). We used mice of two different backgrounds to make sure that the results are not contingent on the specific background: mixed background C57 × FVB-MECP2 duplication mice and 129-MECP2 duplication mice. In order to prove that there are no distinctions, we have analyzed the number of eye movements (EMs) and number of switches per minute during the experiments between two groups. Mixed background mice were produced by crossing C57Bl6J mice to FVB-MECP2 duplication line (Tg1; Collins et al., 2004) mice to generate F1 C57 × FVB-MECP2 duplication mice and nontransgenic littermate controls. Experiments were performed in four- to six-month-old animals. Cohorts were balanced in terms of animal sex (129 background: two male and four female pairs; C57 ×FVB background: four male and three female pairs). There are no significant differences in perception between male and female mice. The experimenters were blind to animal genotypes during experiments and analysis.

### Surgery

All procedures were performed according to animal welfare guidelines authorized by the Brigham and Women’s Hospital IACUC committee. Mice were anesthetized with 1.5% isoflurane. The mouse head was fixed in a stereotactic stage (Kopf Instruments), and eyes were protected with a thin layer of artificial tears ointment (GenTeal). The scalp was shaved and disinfected by applying consecutive swabs of the povidone-iodine solution and 70% ethanol, and then the scalp was resected. A custom-made titanium head plate was attached to the skull with dental acrylic (Lang Dental), preventing occlusion of the mouse’s visual field.

### Visual stimulation

Visual stimuli were generated in MATLAB and displayed using Psychtoolbox (Brainard, 1997). The stimuli were presented on two LCD monitors with a 60-Hz frame rate, positioned ≈10 cm in front of the right eye and covering 180° of the right visual field of the mouse. The screens were γ-corrected, and the mean luminance level was photopic at 80 $\frac{\text{cd}}{{\text{m}}^{2}}$. Visual stimuli consisted of drifting square-wave gratings and plaids of 120° cross angle composed of the grating stimuli components. The gratings had the following parameters: temporal frequency 1.7 Hz, spatial frequency 0.06 cycles/°, spatial duty cycle 0.8 (white bar set to 60%, black bar set to 40%). These parameters were selected to accommodate average spatial frequency and velocity preferences in visually responsive neurons across mouse visual cortical hierarchy (Ohki et al., 2005; Niell and Stryker, 2008; Gao et al., 2010; de Vries et al., 2020). Additive plaid patterns were constructed by summing up component gratings of $50^{{}^{\circ}}$ contrast (Smith et al., 2005). Each instance of plaid or grating movie was preceded by a gray isoluminant screen for 5 min. We kept mean luminance constant throughout both the background and the stimulation periods.

### Optokinetic nystagmus

**Figure 1.**
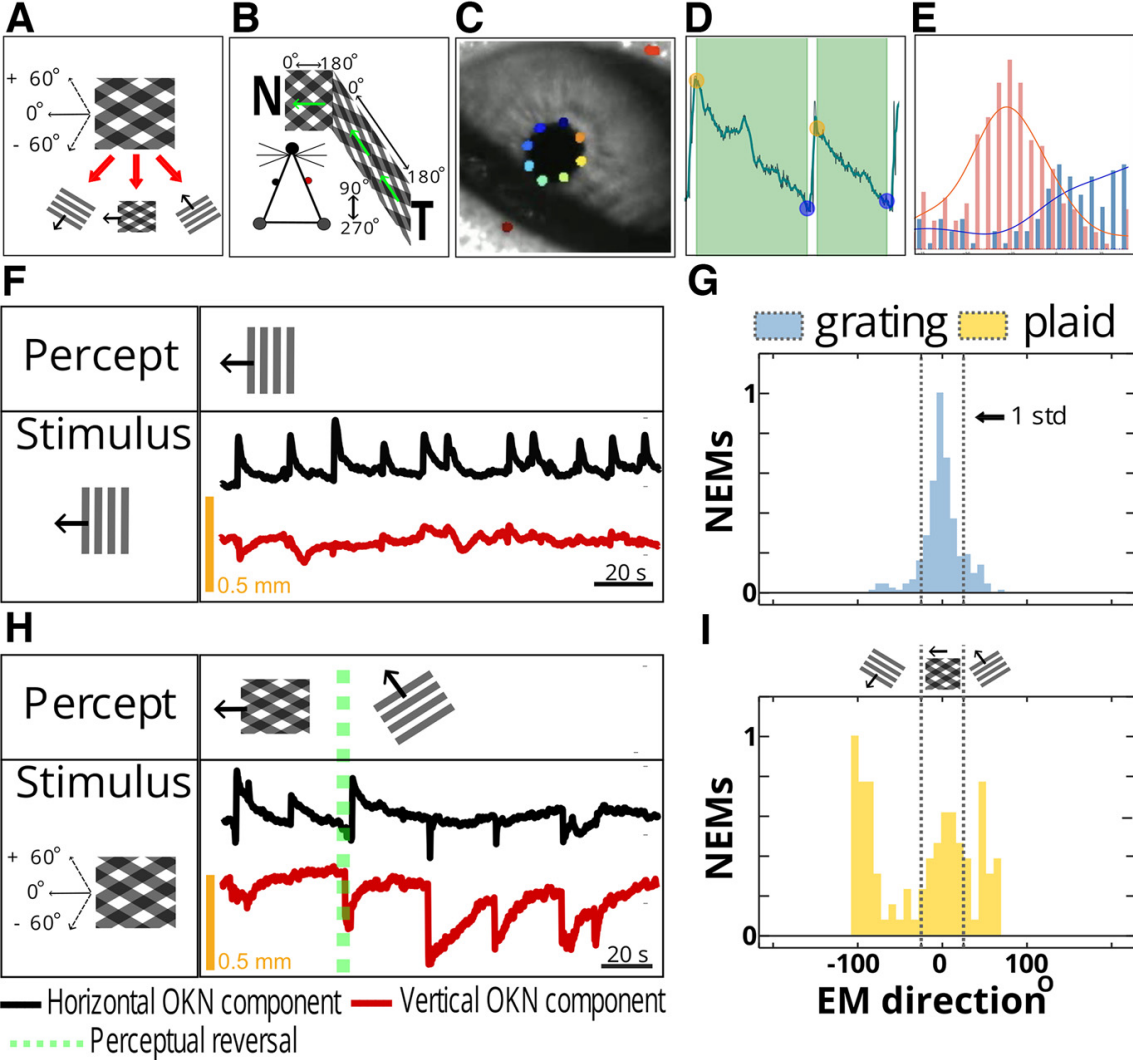
Bistable OKN responses under visual rivalry. ***A***, Bistable moving plaid stimulus. Type I symmetric plaid is composed by summing two 50% contrast component gratings. The gratings move at an angle of 120° relative to each other. This plaid can be seen either as two individual gratings moving at an angle or as a sum of gratings integrated percept of pattern motion. The direction of pattern motion lies in between the directions of motion of each grating. Thus, the observer can follow three directions of motion (lower panel): pattern motion (direction set at 0°) and either of the component grating’s drift, offset at +60° and –60° from the vector of the plaid’s motion (insets). ***B***, Experimental setup. We presented the stimuli on two screens positioned at equal distances from the mouse head to cover 180° of the mouse ipsilateral visual field. We head-posted the mouse to prevent head movements and monitored eye movements with an infrared camera. The mouse could walk freely on the free-moving wheel. Green arrows indicate the direction of the global drift of the stimulus. The stimulus was moving toward the mouse’s nose to induce robust optokinetic movements. ***C***, ***D***, ***E***, Data preprocessing pipeline. ***C***, OKN images were collected at 300 Hz and 20 randomly selected mouse pupil movies were used to train Deep Lab Cut ResNET-150 model to extract the position and size of the animal’s pupil during the OKN (colored dots DeepLabCut feature detection). ***D***, The vertical and horizontal components of the OKN were sorted into saccade-pursuit eye movement pairs, and eye-blink and grooming-related artifacts were located using custom-written Python toolbox “Dolia” and excluded from analysis. ***E***, The pursuit phases of the OKN eye movements were fitted with a linear polynomial fit. The ratio between fitted vertical and horizontal component of each eye movement was then used to determine its direction (angle). Pink and blue histograms show the example distributions of eye movement directions from two different 15-min OKN movies. Using the ratio of the vertical and horizontal components’ amplitudes, the directions of the eye movements were determined (for details, see Materials and Methods). Right, Distributions of the directions of pursuit phases of OKN for two different OKN movies. ***F***, ***G***, Grating-induced OKN. To determine the location of zero direction (pattern motion direction) and classify eye movements as aligned with pattern motion or alternatively the motion of the components, we used OKN data obtained by presenting the zero-direction grating moving in temporonasal direction, similarly to the plaid setup. Since such a grating has only one unambiguous direction of drift, it is possible to use the mean of the eye movement direction distribution as a zero direction. Additionally, [–SD, +SD] can be set as a bracket in which most eye movements aligned with zero direction fall (***E***). The grating-induced OKN is shown in ***F***: as expected, OKN eye movements contain a sole horizontal component (black trace), with no consistent vertical deflections (red trace), and this stimulus does not result in visual rivalry as only one interpretation of the stimulus is possible. In ***G***, the distribution of grating-induced OKN is shown (yellow histogram, 13 zero-direction grating movies from 13 animals were used to determine zero position, and the SD bracket for eye movement classification). [–SD, +SD] interval around the zero direction is then applied to plaid OKN data: the eye movements with directions inside this interval are classified as pattern-motion aligned, while eye movements with directions outside of this interval are classified as component-motion aligned (***G***, blue histogram). ***H***, ***I***, Plaid-induced OKN shows bistable reversals of the eye movement directions. In ***H***, the mouse can follow either the plaid or the grating direction while observing the unchanging plaid stimulus. Green dotted line indicates location of the perceptual switch, defined as the start of the saccade where the animal starts following a different stimulus interpretation. Initially, the animal follows a pattern motion direction; after the reversal, a solid vertical component appears (red trace) as the animal stops following the pattern motion and starts following the +60° component. ***I***, Blue histogram, The EM directions distribution of OKN induced by a plaid stimulus. Gray dotted lines correspond to the pattern-component OKN bracket derived from grating OKN data (see the yellow histogram in ***G***). Plaid OKN: central peak corresponding to pattern-motion aligned eye movements as well as two additional peaks located at approximately +60° and –60° off the central peak and corresponding to component motion-aligned OKN.

We recorded optokinetic eye movements (EMs) elicited during observation of drifting gratings and plaids in 13 head-posted mice MECP2 duplication-littermate pairs. Seven pairs were C57 ×FVB mixed background mice and six pairs were 129 background mice. All animals were awake during the experiment. The stimulus was presented on two screens covering 180° of the visual field of the mouse. The center of each screen was located at 10 cm from the mouse (Fig. 1). We used an infrared camera (model MAKO U-29, Allied Vision Technologies) and a hot mirror to record the movements of the right eye at 300 Hz. We analyzed 5- to 15-min-long movies off-line with Deep Lab Cut toolbox (Mathis et al., 2018) to detect the pupil and extract its diameter and position. Optokinetic eye movement is composed of smooth pursuit following the motion of salient features in the stimulus, followed by a rapid saccade in the direction opposite to the direction of the global stimulus drift to stabilize the image on the retina (Cahill and Nathans, 2008). This pattern of movements (slow pursuit phase plus rapid saccade phase) repeats as long as the stimulus (drifting grating or plaid) is present and is attended by the animal. In order to analyze the acquired time-series data, we have developed a Python 3-based semiautomated software that is available online https://github.com/mecp2-project/Dolia. We analyzed both vertical and horizontal EM components to classify plaid-induced optokinetic nystagmus (OKN) as aligned with local motion percept versus aligned with global motion percept. Periods containing eye-blink artifacts and mouse grooming, that the Deep Lab Cut algorithm identified as having a probability of being a pupil below 95%, were removed from the analysis. We applied a linear fit to the slow pursuit phase of each EM and calculated the eye movement amplitude from each fit (Fig. 1*C*). The direction of each EM was determined by comparing the amplitude of horizontal and vertical saccade projections of EM components. We then plotted histograms of the directions of EMs around $0^{{}^{\circ}}$, which corresponds to the horizontal direction (the direction of the drift of the global stimulus; see Fig. 1). For the plaid-induced OKN, we classified each EM as component or pattern aligned. For this, we first determined the horizontal direction and the average width of the distribution of EM angles evoked by horizontally drifting gratings. We used 1 SD from the mean as the threshold for pattern-aligned EM angles. Thus, any EM whose angle exceeded this threshold was classified as component-aligned, while EMs with angles inside the [−SD,+SD] interval are classified as pattern motion-aligned (Fig. 1*E*,*G*). To study the dynamics of OKN alternations between following the global pattern motion or following component motion, we analyzed two to six 15-min movies of OKN induced by the plaid stimulus moving in the temporonasal (T →N) direction. We extracted the periods of the stable OKN (at least two saccade-pursuit pairs, occurring without a break between the pairs, e.g., saccadic movement is followed by the pursuit phase of the next pair). To identify periods without the OKN (breaks), we first examined the distribution of lengths of pursuits of individual nystagmoid eye movements. Periods of eye drift without return saccades exceeding the 95th percentile of this distribution of lengths were considered breaks in the OKN. During breaks mouse either was not attending to the stimulus and thus experienced no OKN, closed eyes, or experienced eye blinks and grooming bouts. Periods of OKN between breaks (OKN epochs) had to contain at least two consecutive saccade-pursuit pairs to be accepted for the analysis of perceptual reversals. Each movie had to contain at least 3 min of OKN to be accepted for the analysis.

We determined the following parameters:

1. Perceptual reversal rate in each OKN epoch. The rates were averaged over epochs and movies to obtain a median value per animal.
2. The probability of experiencing a switch within 1 min of the beginning of bistable OKN.
3. The durations of “coherent” and “transparent” OKN periods in each animal. Durations were averaged over movies and animals to obtain one median value per animal.
4. Fraction of eye movements aligned with pattern and component motion across all movies of a specific animal.
5. Fraction of OKN epochs with no observed perceptual reversals (nonreversal OKN epochs).

### Statistical tests

Comparing the per-animal reversal rates, dominance period durations, and component/pattern motion ratio we used paired Wilcoxon signed rank (WSR) test comparing the MECP2-duplication mouse to his littermate. We used littermates to control for as many variables as possible and to ensure that the observed effects are truly because of MECP2-ds (S.J. Robertson et al., 2019). Since they share a similar genetic background and are exposed to the same conditions *in utero* and often in early life as well, it makes them ideal controls as it minimizes differences because of genetics and early environmental exposure, which could otherwise confound the results. We have also used paired tests since they have more statistical power than unpaired tests because they control for individual differences between pairs. This ensures that differences observed between groups are because of the condition, rather than individual variation. Statistics were computed across animals. The distributions of switch rates per OKN epoch and durations of dominance periods were fitted with the γ distribution function. To accept or reject the fit for the γ distribution fitting of dominance duration periods and switch rates, we used the $\chi^{2}$ test. All the statistical results can be seen in Table 1.

**Table 1:** Statistical table

Figure	*p*-value	*z* score	*r* (effect size)	*W* (Wilcoxon)
2*A*	0.0134	2.41	0.669	80
2*C*	0.0142	2.377	0.66	79.5
2*D*	0.0027	−2.8	−0.7755	5.5
3*A*	0.0017	2.9	0.8044	87
3*B*	0.0342	−2.12	−0.587	12
3*C*	0.0081	−2.551	−0.7075	9
3*E*	0.0161	−2.353	−0.653	9
3*D*	0.1677 (not significant)			
3*F*	0.6848 (not significant)			

## Results

### Report-free bistable perception paradigm

A reliable way to infer the perceptual state when a bistable visual motion-based stimulus is presented is to measure the direction of the optokinetic nystagmus elicited by the different directions of drift generated by the rivaling stimuli (Enoksson, 1963; Fox et al., 1975; Logothetis and Schall, 1989; Leopold et al., 1995; Wei and Sun, 1998; K. Watanabe, 1999; Naber et al., 2011). Unambiguous fully coherent full-field moving visual stimuli, such as dot fields, coherently moving natural scenes and high-contrast drifting gratings, induce optokinetic nystagmus (OKN) reflex in vertebrate animals such as mammals, birds and fish (Cahill and Nathans, 2008). The OKN is required for the stabilization of retinal input under the conditions of a drifting visual environment. OKN eye movements consist of a slow pursuit in the direction of the stimulus followed by a fast saccade returning the eye to its initial position. OKN has been extensively validated as a reliable indicator of the dominant percept in experimental designs involving ambiguous stimuli, such as binocular rivalry (Fox et al., 1975; Logothetis and Schall, 1989; Wei and Sun, 1998; K. Watanabe, 1999; Naber et al., 2011). Under ambiguous visual conditions, the direction of pursuit during slow phases of OKN is aligned with the direction of motion of the dominant percept (Palagina et al., 2017).

It has been previously shown that mice can exhibit visual bistable perception when exposed to a moving transparent additive plaid stimulus covering ≈270° of the visual field (Palagina et al., 2017). The symmetric transparent additive plaid we used is composed of two transparent gratings of equal contrast and velocity moving at an angle to each other. Under the range of bi-stability promoting stimulus properties, the subjective perception of this stimulus alternates between the “transparent” interpretation, where two full-field component gratings slide on top of each other, and the “coherent” interpretation, where a fused pattern drifting in a direction half-way between the directions of component gratings is seen (Adelson and Movshon, 1982; Moreno-Bote et al., 2010). Large cross-angle between the grating components of the plaid, “transparency-promoting” intersection luminance values of the dark bars (equal to the sum of the luminances of the components), high component grating velocity, asymmetric intersections (occurring when the cross angle between component gratings is above or below 90°) promote a transparent interpretation (Movshon et al., 1985; Moreno-Bote et al., 2010). Symmetry in component gratings’ contrast, spatial frequency and velocity favor the coherent percept (Adelson and Movshon, 1982; Yo and Demer, 1992). Previously, using stimuli fulfilling these criteria (60° or 120° cross-angle between components, contrast normalization, drift velocity 2 cycle/° of visual field, spatial frequency 0.05 cycle/° and symmetry in the properties of component gratings) bistable OKN in C57 wild-type mice were successfully elicited. These properties were tailored to be optimal for mouse area V1 (Ohki et al., 2005; Niell and Stryker, 2008; Gao et al., 2010). In the present study we modified the stimulus keeping in mind the necessity to drive as a large proportion of neurons as possible in different visual areas, which have varying preferences for the drift velocity and spatial frequency of the stimuli. To do this, we changed the duty cycle of the stimuli to 0.8 cycle/° and drift velocity to 1.7 cycle/°, while keeping the components symmetric (spatial frequency 0.06 cycle/°) and contrast normalized to achieve transparency-promoting luminance of the intersections. We used 120° CA (cross-angle) plaids as this was shown to induce an equidominant state (where the observer spends nearly equal time on transparent and coherent percepts) in both human observers (Moreno-Bote et al., 2010) and mice (Palagina et al., 2017). We also reduced the coverage of the visual field to 180° of the right eye’s visual field, as this was shown to induce reliable OKN in mice (Cahill and Nathans, 2008) while allowing us to combine the behavioral task with two-photon imaging or electrophysiological recordings in future experimental work.

Under the updated conditions we show that both 129-background and C57 ×FVB mixed background mice show bistable optokinetic nystagmus, aligned either with the direction of component gratings or the direction of coherent pattern motion (Fig. 1), similarly to what has been observed in C57 mice previously (Palagina et al., 2017). We observed no difference in the rate of generation of OKN between littermates and MECP2 duplication mice or in the magnitude of eye movements (eye movement amplitude, arbitrary units: littermates, 7.43 ± 0.5, MECP2-ds, 6.33 ± 0.51; p=0.308, WSR; OKN rate $\left(\text{in }\frac{\text{eye movement}}{\text{min}}\right)$: littermates, 9.7 ± 1.7, MECP2-ds, 7 ± 0.75, p=0.216, WSR). There was no difference between 129 background animals and C57 ×FVB background animals in terms of OKN properties and dynamics of visual rivalry, thus these two groups were pooled together. The experimental setup is shown in Figure 1. Stimuli were presented on two contiguous screens covering 180° of the mouse contralateral visual field, and pupil position was recorded with the help of hot mirror and an infrared camera. Figure 1*F* shows an example of OKN elicited by a vertically oriented grating moving from the temporal to nasal direction. In this case the eye movements elicited by the stimulus are aligned with the horizontal direction (0°, taken along the temporal →nasal direction). In contrast to the unambiguous horizontally drifting gratings, OKN eye movements elicited by a 120° CA plaid show a tri-modal distribution of eye movement directions: a considerable fraction of eye movements is aligned with one of the two component grating directions in addition to the horizontally aligned OKN that corresponds to the fused pattern motion percept (Fig. 1*H*). This strongly suggests that the perception of the mouse alternates between pattern and component motion for our stimuli in the recorded cohort of mice.

### MECP2 duplication mice show reduced rate and probability of perceptual reversals

**Figure 2.**
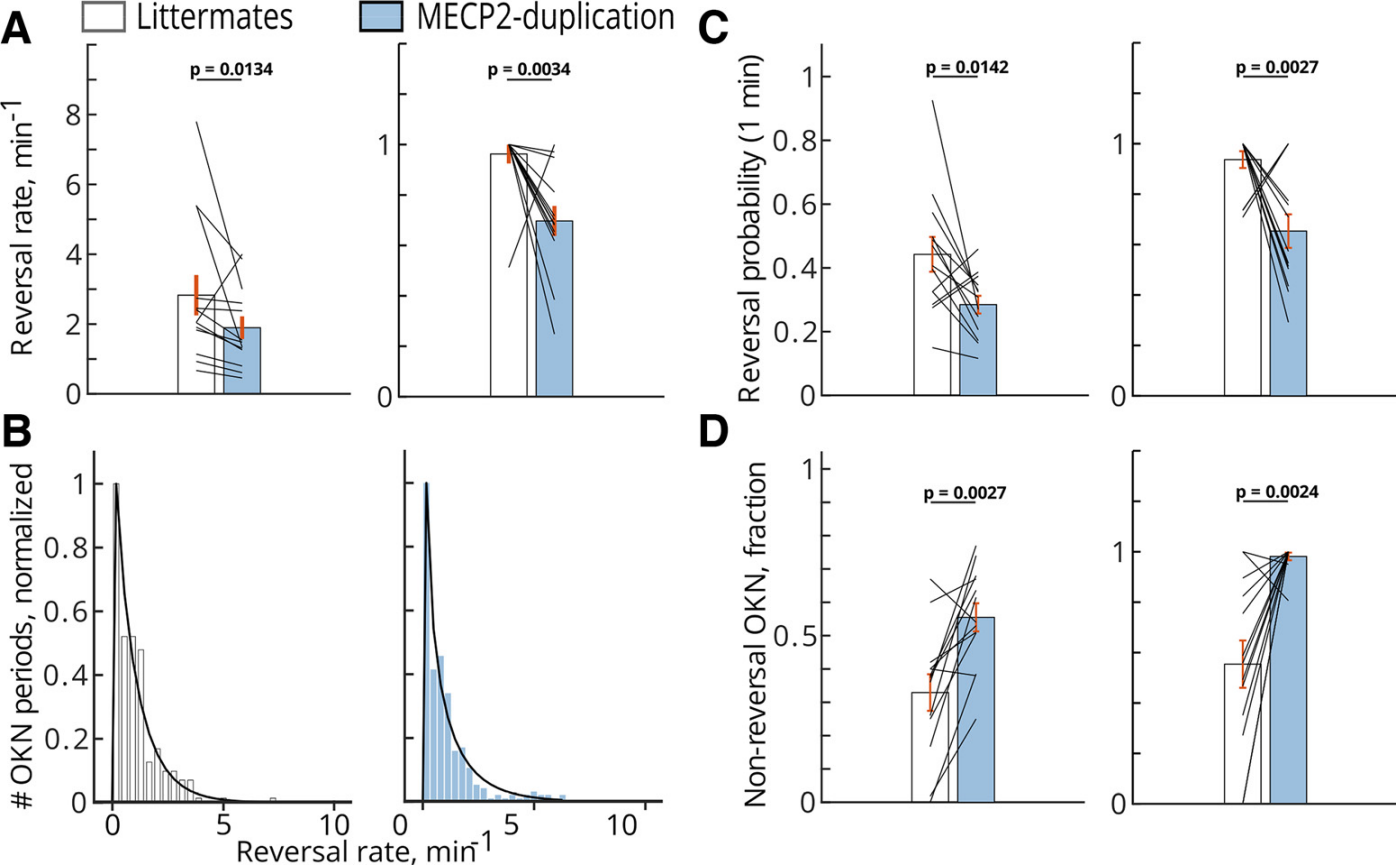
MECP2 duplication syndrome results in reduced perceptual reversal rate during visual rivalry. White bars, littermates; blue bars, MECP2 duplication syndrome. ***A***, The reversal rate (per minute of OKN) is consistently lower in MECP2-ds than in normal littermates. Left panel, Raw data. Right panel, Data normalized by maximum inside each littermate, MECP2 duplication pair. Reversals per minute: littermates, mean ± SEM: 2.8 ± 0.58, median: 2.05; MECP2-ds, mean ± SEM: 1.895 ± 0.325, median: 1.485. ***B***, The distribution of perceptual reversal rates of individual OKN periods. Left panel (white bars), Littermates. Right panel (blue bars), MECP2 duplication. The distributions follow γ distribution fit (littermates: p<0.0001; MECP2-ds: p<0.0001, $\chi^{2}$ test). Data were pooled across OKN periods belonging to 13 littermates and 13 MECP2 duplication animals, respectively. Before pooling, each animal’s dataset was normalized by its mean rate. ***C***, In accordance with the reduced reversal rate in MECP2 duplication, the probability of observing a switch after 1 min of ongoing plaid-induced OKN was also reduced in MECP2 duplication mice. The left panel indicates raw data, while the right panel shows the data normalized by maximum inside each littermate, MECP2 duplication pair. Reversal probability: littermates, mean ± SEM: 0.4425 ± 0.054, median: 0.407; MECP2-ds mean ± SEM: 0.284 ± 0.028, median: 0.308. ***D***, MECP2 duplication mice consistently show a substantial fraction of OKN periods where no reversals occur, and the animal persistently tracks either pattern (“coherent” percept) or component direction (“transparent” percept). Left panel, Raw data. Right panel, Data normalized by maximum inside each littermate, MECP2 duplication pair. Nonreversal OKN fraction: littermates, mean ± SEM: 0.33 ± 0.055, median: 0.374; MECP2-ds, mean ± SEM: 0.555 ± 0.04, median: 0.54. All *p*-values are determined by two-sided Wilcoxon signed rank (WSR) test, n=13 pairs. Wilcoxon test statistic, *p*-values, *z*-scores and effect sizes for panels ***A***, ***C*** and ***D*** are reported in Table 1.

We next examined the dynamics of bistable reversals between “coherent” interpretation OKN (mouse tracking global pattern; OKN eye movements aligned with the global pattern direction) and “transparent” interpretation OKN (mouse tracking the component gratings; OKN eye movements aligned with the direction of drift of either component grating) in MECP2 duplication animals versus unaffected littermates. Both MECP2 duplication animals and littermates displayed bistable reversals. However, in MECP2 duplication syndrome mice the rate of reversals was reduced compared with their normal littermate pairs (Fig. 2*A–C*), and MECP2 duplication animals displayed more frequent OKN epochs where only a single interpretation of the stimulus was consistently followed and no perceptual reversals occurred (Fig. 2*D*; nonreversal OKN fraction: littermates, mean ± SEM: 0.33 ± 0.055, median: 0.374; MECP2-ds, mean ± SEM: 0.555 ± 0.04, median: 0.54; p=0.0027, WRS). Consequently, the fraction of OKN epochs showing bistable reversals was reduced in MECP2 duplication animals. Littermates showed on average 2.8 reversals per 1 min of OKN movie (mean ± SEM: 2.8 ± 0.58, median: 2.05), while MECP2-ds mice showed 1.9 reversals per minute (mean ± SEM: 1.895 ± 0.325, median: 1.485), a significant reduction in bistable reversal rate ( p=0.0134, WSR, n=13 pairs; Fig. 2*A*,*B*). The probability to observe a switch after 1 min of uninterrupted plaid-driven OKN was consequently reduced in MECP2 duplication mice (littermates, mean ± SEM: 0.4425 ± 0.054, median: 0.407; MECP2 duplication mean ± SEM: 0.284 ± 0.028, median: 0.308; p=0.0142, WSR; Fig. 2*C*). In sum, the properties of bistable reversal dynamics are altered in MECP2 duplication mice, with duplication animals showing increased proportion of reversal-free OKN epochs and reduced reversal rate and probability.

### Local versus global motion processing in MECP2 duplication mice and increased stability of local motion “transparent” percepts

**Figure 3.**
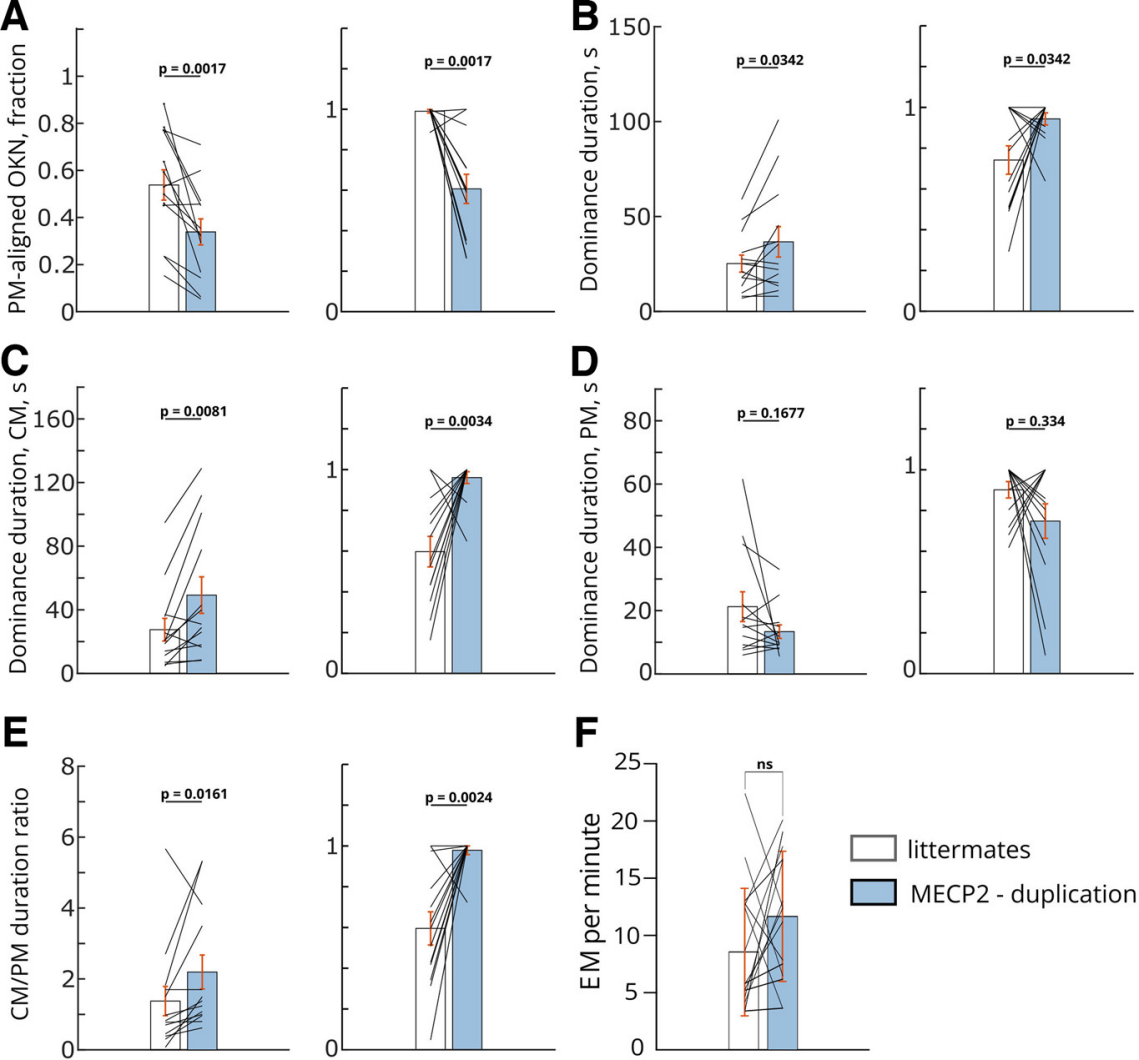
Atypical preference for local motion processing in MECP2 duplication syndrome. The reduced rate of perceptual reversals in MECP2 duplication mice is driven by the lengthening and overstability of component motion (“transparent”) percepts. CM, component movement; PM, pattern movement. ***A***, The total fraction of nystagmoid eye movements aligned with pattern motion direction (“coherent” percept). Although there is considerable variance across data, in normal littermates (clear bar), nearly equal fractions of eye movements are aligned to either pattern motion direction (“coherent” percept, global motion) or component motion direction (“transparent” percept, local motion). In contrast, in MECP2 duplication mice, a greater portion of OKN eye movements is allocated to component local motion, and the fraction of pattern motion-aligned eye movements is reduced. Littermates, mean ± SEM: 0.538 ± 0.064, median: 0.53; MECP2-ds, mean ± SEM: 0.339 ± 0.055, median: 0.326. Left panel, Raw data. Right panel, Data normalized by maximum inside each littermate, MECP2 duplication pair. ***B***, Dominance duration is increased in MECP2 duplication mice, following the decrease in reversal rate and reversal probability (Fig. 2). Littermates, mean ± SEM: 25.2 ± 4.5, median: 21; MECP2-ds, mean ± SEM: 36.7 ± 8, median: 25. Left panel, Raw data. Right panel, Data normalized by maximum inside each littermate, MECP2 duplication pair; *p*-values, WSR. ***C***, ***D***, The increase in average dominance duration is carried mainly by the increased durations of “transparent” percepts when the mouse is following the local motion of component gratings (***C***, littermates, mean ± SEM: 27.5 ± 7.11, median: 20.2; MECP2-ds, mean ± SEM: 49.2 ± 11.5, median: 31), while the global motion “coherent” percepts show inconsistent changes with shortening in some animals and lengthening in others (***D***, littermates, mean ± SEM: 21.2 ± 4.7; MECP2-ds, mean ± SEM: 13.36 ± 2.2). As a result, although there is a general trend of shorter pattern-motion percepts in MECP2 duplication mice, it is not significant ( p=0.1677). Left panels, Raw data. Right panels, Data normalized by maximum inside each littermate, MECP2 duplication pair. ***E***, The ratio of dominance period durations is shifted in favor of transparent local motion percepts at the expense of global motion “coherent” percepts. Littermates, mean ± SEM: 1.376 ± 0.41, median: 0.82; MECP2-ds, mean ± SEM: 2.2 ± 0.48, median: 1.38. Left panel, Raw data. Right panel, Data normalized by maximum inside each littermate, MECP2 duplication pair. ***F***, The number of eye movements per minute in WT and MECP2-duplication mice. Littermates, mean ± SEM: 11.38 ± 1.81, median: 12.37; MECP2-ds, mean ± SEM: 9,07 ± 1.41, median: 7.54. These results indicate that the difference in frequency of eye movement is not significant ( p=0.6848). Left panel, Raw data. Right panel, Data normalized by maximum inside each littermate, MECP2 duplication pair. White bars, Littermates. Blue bars, MECP2 duplication. All *p*-values are determined by two-sided Wilcoxon signed rank (WSR) test, unless noted otherwise, n=13 pairs. Wilcoxon test statistic, *p*-values, *z*-scores and effect sizes for panels ***A-F*** are reported in Table 1.

The slower rate of rivalry in MECP2 duplication mice was accompanied by pronounced changes in the processing of visual motion. Specifically, MECP2 duplication animals showed strong preference for the component motion compared with their normal littermates (Fig. 3). The latter either spent approximately equal time following component gratings versus coherent pattern direction, or showed preference for coherent pattern direction. This effect was seen both in the total fraction of OKN eye movements aligned with the component versus the pattern directions (Fig. 3), and in the duration of component-versus pattern-dominance periods (Fig. 3*B*,*C*). Interestingly, for dominance periods, the strongest effect was observed in the duration of component percepts, which were on average twice as long in MECP2 duplication animals as in littermate controls (Fig. 3*C*, littermates, mean ± SEM: 27.5 ± 7.11, median: 20.2; MECP2-ds, mean ± SEM: 49.2 ± 11.5, median: 31). This effect was highly reproducible across pairs and highly significant (Fig. 3*C*, p=0.0081, WSR). In contrast to the component-aligned OKN periods, the durations of pattern motion-aligned OKN periods showed disparate effects: in some duplication-littermate pairs MECP2 duplication led to an increase in pattern percept durations, while in other a decrease was observed (Fig. 3*D*). Pooled data, including both durations of component and pattern motion-aligned OKN showed a net increase in dominance period durations, consistent with a reduced rate of perceptual reversals (Fig. 3*B*, littermates, mean ± SEM: 25.2 ± 4.5, median: 21; MECP2-ds, mean ± SEM: 36.7 ± 8, median: 25; p=0.0342, WSR). In addition, MECP2 duplication animals showed a consistent shift of the ratio between component motion percept duration and pattern motion percept duration in favor of component motion percepts (Fig. 3*E*). These findings imply that the bulk of the effect that MECP2 duplication has on the perceptual reversals occurs because of increased stability of the component motion “transparent” percepts and a resulting shift of the ratio between component-pattern motion percept duration in favor of the component (“transparent”) interpretation. Ultrastable component motion percepts then may contribute to lower probability to observe a reversal.

## Discussion

### Slower dynamics of visual rivalry in MECP2 duplication syndrome

We view the world as generally stable even in the face of fast dynamic changes, such as fast-moving objects and emerging stimuli. This stability rests on an uncertain foundation: naturalistic scenes are inherently ambiguous, and the stable percepts of them are a result of a probabilistic process reflecting the most likely interpretation of the inputs. As a result, neuronal populations are constantly engaged in such ongoing interpretation and adjust their decision variables accordingly (Leopold and Logothetis, 1999; Sterzer et al., 2009; Aggelopoulos, 2015). In bistable and multistable perception, the competing interpretations of the sensory input cannot ultimately win against each other; as a result, the brain vacillates between the conflicting interpretations although the stimulus stays the same. Visual rivalry involves a network of areas spanning V1, visual association areas, frontal lobe, supplementary motor cortex, and prefrontal cortex (Leopold and Logothetis, 1996; Kleinschmidt et al., 1998; Lumer et al., 1998; Lumer and Rees, 1999; Knapen et al., 2011). As a result, top-down cortical processes stemming from sensory-motor integration, attention and decision-making affect the dynamics of visual rivalry. Perception, decision-making, and cognate sensory processing are pervasively impacted in neurologic circuitopathies such as schizophrenia and autism (C.E. Robertson et al., 2013; Schmack et al., 2015; Heeger et al., 2017). Specifically, in idiopathic human autism, atypical sensory perception co-exists with higher-order deficits in social communication, cognitive flexibility, and executive function (Simmons et al., 2009; American Psychiatric Association, 2013; C.E. Robertson and Baron-Cohen, 2017; Van der Hallen et al., 2019). As a distributed computation involving both the low-level sensation and perception processes and high-level processes pertaining on attention and decision-making, visual rivalry emerges as an attractive paradigm to study these processes and their interaction in the autism spectrum. In our study, we applied a monocular rivalry paradigm to explore whether the dynamics of bistable visual perception were affected in the mouse model of MECP2 duplication syndrome of autism (Collins et al., 2004). This model reproduces some features of human autistic syndromes, including enhanced motor learning, motor, and visual stereotypies, and increased likelihood of seizure events (Collins et al., 2004; Samaco et al., 2012; Jiang et al., 2013; Sztainberg et al., 2015; Ash et al., 2017, 2021a, b, 2022; Zhang et al., 2017; Zhou et al., 2019). We found that the rate of perceptual reversals is decreased (Fig. 2) in MECP2 duplication syndrome, while the average duration of individual percept dominance periods is prolonged (Fig. 2). These effects occurred regardless of the genetic line background of the mice, since both 129-MECP2 duplication line and FVB*C57 mixed background duplication line have been used. Reduced rate of perceptual reversals under visual rivalry conditions in MECP2 duplication mice recapitulates the phenotype occurring in human idiopathic autism. (C.E. Robertson et al., 2013; Spiegel et al., 2019). The magnitude of this reduction correlates with the expression of other autistic core traits, such as the severity of social communication deficits and ADOS score (Spiegel et al., 2019). Furthermore, in autistic subjects, slower binocular rivalry shares a common anatomic substrate with general cognitive rigidity, a part of the core repetitive restricted behaviors and interests (T. Watanabe et al., 2019).

### Atypical perception of visual motion in MECP2 duplication syndrome

Enhanced attention to visual detail and superior processing of local visual information are core traits of autism. Specifically, in autism, the visual perception is superior when the task is based on detecting local elements in the visual scene while the performance suffers when the subjects must focus on global elements (Shah and Frith, 1983; Jolliffe and Baron-Cohen, 1997; Plaisted et al., 1998, 1999; Mottron et al., 1999; Rinehart et al., 2000; Happé et al., 2001; Jarrold et al., 2005; C.E. Robertson and Baron-Cohen, 2017). This perceptual phenotype is usually described in literature as “not seeing the forest behind the trees” (Frith, 2003; C.E. Robertson et al., 2012). Of particular relevance to our study are autism-related changes in the processing of visual motion and integration of local moving cues into a global moving percept (Bertone et al., 2003; Pellicano et al., 2005; Kaiser and Shiffrar, 2009; Brieber et al., 2010; Koldewyn et al., 2010; C.E. Robertson et al., 2012, 2014; Van der Hallen et al., 2019). The bistable perception paradigm in our study makes use of two competing interpretations of a moving plaid: (1) the “transparent” interpretation where component gratings are seen as separate stimuli moving on top of each other, and (2) the “coherent” interpretation, where the stimulus is seen as a fusion of two moving component gratings resulting in a percept of moving pattern (Adelson and Movshon, 1982; Castelo-Branco et al., 2000; Smith et al., 2005). It is proposed that processing of complex stimuli like moving plaid rests on two distinct populations of neurons: orientation-selective and direction-selective component neurons and direction-of-motion selective pattern cells. While the first specialize on local motion information processing and responding to individual moving grating components, the latter ignore the orientation of the grating components, and instead respond to any stimulus moving in the preferred direction, including large-sized moving patterns such as naturalistic moving visual scenes. Pattern motion selectivity is posited to arise by integrating the inputs from component-motion-sensitive neurons. As one moves from primary visual areas to more specialized areas of the visual dorsal stream, the fraction of pattern cells and neurons integrating various types of local sensory information and computing global motion increases (Rodman and Albright, 1989; Gizzi et al., 1990; Albright and Stoner, 1995; Movshon and Newsome, 1996; Scannell et al., 1996; Smith et al., 2005; Rust et al., 2006; Khawaja et al., 2009; Juavinett and Callaway, 2015; Palagina et al., 2017). Pattern-motion processing in lower-order visual areas like primary visual cortex V1 is strongly dependent on feedback from higher-order areas (Guo et al., 2004), while the integration of local motion cues into the global moving scenes by higher-order areas depends on the feedforward inputs from the V1 (Movshon et al., 1985).

Therefore, our competing interpretations are based on categorically different subtypes of visual motion: (1) local motion (when two individual component gratings are seen) and (2) global motion, occurring via integration of local motion cues and subsequent fusion of two gratings into a global moving pattern (as occurs in coherent moving plaid interpretation). Moreover, these two processes (global vs local motion) are linked by feedforward and feedback connections across the cortical hierarchy.

In MECP2 duplication mice, we observed a pronounced preference for local motion percepts, both in terms of the fraction of eye movements aligned with component gratings and in terms of the duration of transparent versus coherent percepts (Fig. 3). This recapitulates the visual motion processing peculiarities found in a subset of human subjects with autism (C.E. Robertson and Baron-Cohen, 2017; Van der Hallen et al., 2019). Namely, studies using random dot kinematogram (RDK) display a subset of subjects with autism show increased motion coherence thresholds (e.g., a larger fraction of dots have to move together in the specified direction for the subject to detect coherent motion). However, this difference diminishes and disappears when the decision window is extended, implying that integration of local moving cues into a global moving percept is slowed down, but not fundamentally impaired or absent in autism spectrum (C.E. Robertson et al., 2014). Another group of studies found no differences in the behavioral performance of subjects when viewing RDK displays; however, subjects with autism still showed differential activation of visual areas in the dorsal stream, such as primary visual cortex (V1) and human middle temporal complex (hMT), while observing and reporting coherent motion (Brieber et al., 2010; Van der Hallen et al., 2019). In a similar vein, our MECP2 duplication mice still consistently experience global moving pattern percepts. However, their durations show inconsistent changes: longer in one subgroup of MECP2 duplication animals and shorter in the others. While the duration of transparent percepts relying on local motion processing is consistently and dramatically increased compared with normal littermates (Fig. 3).

### Interaction between the atypical perception of visual motion and reduced rate of perceptual reversals

In our paradigm, the MECP2 duplication mice show prolonged dominance periods of local motion perception. In contrast, the global motion percepts are generally shortened or unchanged, leading to shifted motion processing ratio favoring the local motion information over integrated motion information (Fig. 3). Additionally, the total fraction of OKN eye movements aligned with component motion is greatly increased in MECP2 duplication, while the OKN fraction aligned with pattern motion is reduced (Fig. 3). These observations imply that the capacity of neuronal populations reserved for the global motion percept formation and/or maintenance is reduced in MECP2 duplication syndrome, or the dynamics of such integration are altered. This is in line with two theories of autism, dorsal stream deficit theory (Braddick et al., 2003; Macintyre-Beon et al., 2010; Greenaway et al., 2013; Chieffi, 2019) and weak central coherence theory (Happé et al., 2001; Dakin and Frith, 2005; Happé and Frith, 2006). Dorsal stream deficit theory states that circuitry allocated to computing global motion from local moving cues is deficient in autism. In children with autism, this is exemplified by difficulties in following multiple moving objects simultaneously, impaired imitation of visual learning tasks, and performing complex movements without somatosensory feedback, since visual guidance of the motor output is disrupted (Williams et al., 2004; Macintyre-Beon et al., 2010). Weak central coherence, on the other hand, proposes that global motion perception deficit may be because of a general cognitive style that prioritizes fine local details over global features (Happé and Frith, 2006). In both types of explanation, the preference of MECP2 duplication mice for local features at the expense of globally coherent motion may be a major contributor to diminished rate of visual rivalry. The bias for one specific rivaling interpretation of the stimulus may impair the ability of the brain to select an alternative interpretation and thus affect the rate of visual rivalry. In MECP2 duplication, the coherent motion percepts appear to either not amass enough neuronal population activity or synchrony to remain stable, while local-motion percepts gain stability (Fig. 3). Interestingly, the physiological basis for these changes may occur as early as primary visual cortical area V1 (C.E. Robertson et al., 2014; Palagina et al., 2017; Ash et al., 2022). First, pyramidal neurons in area V1 of MECP2 duplication mice show overly reliable firing in response to local motion information (for example, when moving gratings are used as a stimulus; Ash et al., 2022). Second, area V1 harbors a significant portion of visual neurons dedicated to the processing of local motion and, in mice, contributes to the dynamics of bistable perception: removing V1 via lesion causes a decrease in the fraction of component motion-aligned OKN corresponding to local motion percepts (Palagina et al., 2017). In idiopathic human autism, hyperactivation of area V1 was found in a subset of subjects during the processing of coherent motion (Brieber et al., 2010). Additionally, in another subset of subjects with autism the areas of the dorsal stream, including V1 and middle temporal area, showed delayed activity during motion coherence processing (C.E. Robertson et al., 2014). Finally, neuronal responses of MECP2 duplication mice in area V1 show reduced coupling to ongoing cortical activity (Ash et al., 2022). This may result in disruption of both feedforward inputs from V1 to higher-order areas and weakening of the feedback from these higher-order areas to V1, reducing the integration of local motion cues there (Ash et al., 2022). Taken together, these observations point to an interesting possibility that the over-representation of local component motion in area V1 and disrupted connections between V1 and the rest of the visual dorsal stream are major contributors to the reduced rate of visual rivalry in autism. The reason is that they confer an advantage to the local motion information in the moving stimuli. In contrast, the synthesis of local information into the global motion of the scenes becomes impaired.

### Rate of visual rivalry, global motion synthesis, and excitatory-inhibitory balance in cortical circuits

One of the prominent theories in autism states that core traits of the condition occur secondary to altered development of cortical interneurons and resulting shift in the balance between excitation and inhibition in cortical circuits across sensory and higher-order cortical areas (Casanova et al., 2003; Rubenstein and Merzenich, 2003; Gogolla et al., 2009; C.E. Robertson et al., 2014, 2016). Dynamics of visual rivalry and the rate of perceptual reversals are similarly hypothesized to depend on excitation-inhibition circuit wiring in the competing clusters of neurons coding for rivalrous percepts (Laing and Chow, 2002; Klink et al., 2008a,b; Seely and Chow, 2011). Computational models of binocular rivalry show that shifting excitatory-inhibitory ratio causes an increase in dominance durations, as eye-specific inputs maintain stable activity for more extended periods (Dayan, 1998; Wilson, 2003; Klink et al., 2008a,b, 2010; van Loon et al., 2013). Altered local opponent inhibition in visuomotor areas was proposed to underlie the delayed integration of local moving features into global motion percepts in autism (C.E. Robertson et al., 2014). MECP2 dysfunction was shown to alter synchrony and net excitation-inhibition balance in neuronal circuits, with a greater impact on the phenotype of GABAergic interneurons. Overexpression of MECP2 was shown to affect predominantly genes affecting GABAergic signaling (Chao et al., 2010; Cai et al., 2020), with the result of disrupted synchronization within local and brain-wide networks (Shou et al., 2017). Thus, our findings that visual rivalry dynamics are slowed in MECP2 duplication mice and that they favor local motion percepts over global motion percepts are consistent with the altered excitation-inhibition dynamics theory of the autistic brain.

In summary, our MECP2 duplication mice phenotype reproduces core features of the autism spectrum, atypical perception of visual motion and slower dynamics of visual rivalry and thus can serve as a valid model of neural circuit dysfunction. Going forward, our bistable perception paradigm combined with two-photon imaging and optogenetic manipulations (Yizhar et al., 2011; Nikolenko et al., 2013; Sofroniew et al., 2016) in the MECP2 duplication mouse model can be used to directly and causally test the following theories of the autism: excitatory-inhibitory imbalance, weak central coherence, dorsal stream deficiency and disrupted intracolumnar and cortex-wide connectivity.

## Synthesis

Reviewing Editor: Christopher Colwell, UCLA School of Medicine Decisions are customarily a result of the Reviewing Editor and the peer reviewers coming together and discussing their recommendations until a consensus is reached. When revisions are invited, a fact-based synthesis statement explaining their decision and outlining what is needed to prepare a revision will be listed below. The following reviewer(s) agreed to reveal their identity: Baris Alten.

The authors show that mice that overexpress human MecP2 toggle differently (compared to WT littermates) across global coherent and local transparent interpretations of an ambiguous visual stimulus. Specifically, the authors show that, in MeCP2 duplication mice, there are overstable transparent interpretations of the stimulus, and overall slower dynamics of toggles across coherent and transparent.

Major concerns that need to be addressed.

- It’s not clear how many mice have been used. In the experimental procedures, the authors mention 4WT +4KO on the c57-fvb background, and also 3WT + 3KO on the 129 B6 background. Then in the OKN description, they mention 7 pairs (7KO and 7WT) under the C57-fvb, and 6 pairs (6KO and 6 WT) under the 129 B6 background.

- We are concerned about the mixed backgrounds used. Please provide the data supporting the statement that there are no differences across these two backgrounds. For example, the authors could use a statistical mixed model to test whether the background is correlated with the measurements.

- Please provide better justification of comparing the WT and KO mice within the same cage. Are there group differences when the mice are not separated by litters?

- Please indicate in the text that the study was performed in awake mice as this is an important aspect of the study. Is it possible that the dynamics of probabilities of coherent and local interpretations of the ambiguous stimulus could be driven more simply by the temporal pattern of locomotion of the mouse? Are the coherent/transparent probabilities influenced by locomotor activity?.

- Please provide the rationale for the choice of 1 min window to detect the stability of either a coherent or transparent interpretation of the ambiguous stimulus is totally arbitrary. Can the authors compare cumulative distributions of the probabilities of transitions rather than looking at bar graphs set up under an arbitrary threshold?

Minor points:

- The authors mix males and females in the study. Is it possible that the human MeCP2 transgene got inserted into the X chromosome? If so, this should be brought up as an additional confounding factor.

- Add axis names (make it visible) to figure 1 panels D, E, F and H.

-In figures 2 and 3, the lines connect WT and MECP2 littermates, but seems like they represent same subjects with paired measurements. Maybe remove the lines?

-Remove the horizontal axis below the 0 in figures 2 and 3.

-Replace graphic legend (white and blue boxes with WT and MECP2-ds) to either before/above the first panel or below each graph under the appropriate columns instead of keeping at the end of the last panel.

-Double check that each abbreviation has been clearly described in the text and in the figure legends.

## Author Response

Dear members of the reviewing committee,

We would like to express our sincere appreciation for your thoughtful and constructive feedback on our paper. We are truly grateful for your efforts in helping us improve our work. We would like to address each point raised in your comments. In addition, we have generated supplementary figures addressing some of the concerns. These figures are added in the response below; however, if the reviewers feel strongly that they need to appear in the main manuscript, we are happy to incorporate them there.

Major revisions:

1. Reviewer: “It’s not clear how many mice have been used. In the experimental procedures, the authors mention 4WT +4KO on the c57-fvb background, and also 3WT + 3KO on the 129 B6 background. Then in the OKN description, they mention 7 pairs (7KO and 7WT) under the C57-fvb, and 6 pairs (6KO and 6 WT) under the 129 B6 background”.

“We are concerned about the mixed backgrounds used. Please provide the data supporting the statement that there are no differences across these two backgrounds.”

We apologize for the confusion on the number of mice in the manuscript; we have now made the number of mice used in our study more explicit. Specifically, we have included the following information regarding the background of the mice: “129 background: 2 male and 4 female pairs; C57×FVB background: 4 male and 3 female pairs.”

Our intention in using mice with mixed backgrounds was to ensure that the results we obtained were not dependent on a specific background. To alleviate the concern on potential differences between the backgrounds, we have included here supplementary data analyzing the number of eye movements and the number of reversals per minute during the experiments between the two groups (Figure 1).

2. Reviewer: “Please provide better justification of comparing the WT and KO mice within the same cage.”

To control for as many variables as possible and ensure that the observed effects are specifically attributed to MECP2-ds, we used littermates in our study. By utilizing littermates, who share a similar genetic background and are exposed to the same conditions in utero and often during early life, we minimize genetic and early environmental differences that could potentially confound the results. Additionally, we employed paired tests as they offer greater statistical power compared to unpaired tests by accounting for individual differences between pairs. This approach enables us to be more confident that any observed differences between groups are indeed due to the condition under investigation, rather than individual variation. For clarity, we have added this explanation to the manuscript.

3. Reviewer: “Please indicate in the text that the study was performed in awake mice as this is an important aspect of the study. Is it possible that the dynamics of probabilities of coherent and local interpretations of the ambiguous stimulus could be driven more simply by the temporal pattern of locomotion of the mouse? Are the coherent/transparent probabilities influenced by locomotor activity?”

We appreciate your valuable comment and added the sentence to our manuscript stating that the mice were awake during the experiment. Fortunately, we have recorded locomotor activity in some of our mice (6 mecp2-littermate pairs), allowing us to explore the relationship between the reversal onsets and locomotion activity of the mouse. We found minimal to no correlation between the coherent/transparent probabilities and locomotor activity (Figure 2).

4. Reviewer: “Please provide the rationale for the choice of 1 min window to detect the stability of either a coherent or transparent interpretation of the ambiguous stimulus is totally arbitrary. Can the authors compare cumulative distributions of the probabilities of transitions rather than looking at bar graphs set up under an arbitrary threshold?”

We followed the reviewer’s suggestion and built the cumulative probability plots for the dominance durations, shown here in Figure 3, for all animals communally and for each littermate-MECP2-ds pair (two examples shown in Figure 3). It is clear from the communal plot (Figure 3A), that by 1 minute most animals would have reversed at least once. However, it is clear that MECP2-ds cumulative curve displays a shift with longer dominance durations (and thus reduced probability to reverse) across whole range of times, except extremums at zero and longest dominance periods. This is evident in both communal data from whole set (Figure 3A) and example data from littermate-MECP2-ds pairs (Fig.3, B and C).

Thus, 1 minute interval for comparing reversal rates and probabilities was selected based on the fact that ∼90 % of stable percept periods are shorter than 1 minute.

Minor revisions:

1. We have compared male and female mice and determined that there are no significant differences in OKN properties between the two sexes.

2. Regarding the panels in question, we understand your concern. However, please note that these schematic panels D and E are intended to provide a visual understanding of our work pipeline. Consequently, we are somewhat hesitant to include axes in those panels to avoid potential confusion. We have added Y-axes to panels F and H and described the scale bars properly. We apologize for missing this previously.

3. We acknowledge your point about the lines in the graph. If we were to remove the lines, it would become unclear which points represent a MECP2-Littermate pair, and our statistical analyses were done across pairs and using paired tests. Hence, we have opted to retain the lines to maintain clarity in the graphical representation.

4. We have taken your feedback into consideration and have relocated the graphic legend to the top/before the panels where it was possible for improved readability. Furthermore, we have thoroughly reviewed and double-checked the figure legends and abbreviations to ensure accuracy and clarity.

Once again, we very much appreciate your valuable input, and we hope that the revisions we have made address your concerns. We remain committed to enhancing the

quality of our paper based on your suggestions.
